# Does the relationship between stress and quality of life differ among informal caregivers of older adults with Alzheimer’s disease and children with autism spectrum disorder? Results from a cross-sectional survey

**DOI:** 10.1186/s41687-025-00953-7

**Published:** 2025-10-16

**Authors:** Nilesh Gangan, Meagan Rosenthal, Arman Arabshomali, Erin Holmes, Benjamin F. Banahan, Ruchit Shah, John P. Bentley

**Affiliations:** 1 Carelon Research, Wilmington, DE USA; 2 Department of Pharmacy Administration, University of Mississippi School of Pharmacy, University, MS USA; 3 Center for Pharmaceutical Marketing and Management, University of Mississippi School of Pharmacy, University, MS USA; 4 Daiichi Sankyo, Basking Ridge, NJ USA

**Keywords:** Informal caregiver, Quality of life, Stress, Austism spectrum disorder, Alzheimer’s disease, Caregiver-reported outcomes, Structural equation modeling

## Abstract

**Background:**

Informal caregiving may impact the physical, mental and economic well-being and ultimately the quality of life (QOL) of the caregivers, however, each caregiver may differ in the degree to which their QOL is impacted. Little is known about association of stressors and coping mechanism in subjective variation of QOL among informal caregivers. This study aimed to understand the appraisal process for QOL among informal caregivers and compare informal caregivers of older adults and children with respect to their appraisal of QOL.

**Methodology:**

This cross-sectional study utilized a web-based survey to gather data from two distinct groups of informal caregivers: those caring for older adults with Alzheimer’s Disease (AD) and those caring for children with Autism Spectrum Disorder (ASD). Caregivers at least 18 years of age were administered a survey that included previously-developed scales to measure stress, coping mechanism, and QOL. Structural equation modeling was employed to assess the mediating role of coping behaviors in the relationship between perceived stress and QOL and multiple-group analysis was performed to evaluate differences between the caregiver groups.

**Results:**

Of the 417 included caregivers, 210 (50.4%) were AD patient caregivers and 207 (49.6%) were ASD patient caregivers. In model testing, perceived stress was negatively associated with global QOL for both groups (i.e. direct effects) (estimate = −0.637, 95% CI: −0.777 to −0.529 for the ASD caregiver group; estimate = −0.601, 95% CI: −0.731 to −0.468 for the AD caregiver group). The only statistically significant indirect effect was perceived stress on global QOL through dysfunctional coping for the AD caregivers (estimate = −0.026, 95% CI: −0.048 to −0.005). None of the differences in indirect and direct effects between the two caregiver groups were statistically significant.

**Conclusions:**

Perceived stress was significantly associated with QOL among informal caregivers, irrespective of the condition of the patient cared for, be it AD or ASD. Dysfunctional coping strategies played a significant role in mediating this relationship only among AD caregivers. However, the two caregiver groups did not differ between them in appraisal of QOL. Stress management among caregivers will aid in their ability to have a good QOL.

**Supplementary Information:**

The online version contains supplementary material available at 10.1186/s41687-025-00953-7.

## Introduction

Individuals with chronic physiological or psychological conditions accompanied by functional or cognitive limitations, often need assistance with activities of daily living. In such cases, their adult children, parents, spouses/partners, or friends are well-positioned to provide efficient and inexpensive care compared to formal caregiving, i.e., hiring a paid nurse or being in a daycare facility. According to a report from The National Alliance for Caregiving, an estimated 53 million adults provide informal caregiving to an adult or a child in the United States (US) [1]. The costs saved from care provided by informal caregivers is significant, estimated to be roughly $600 billion, with an average care provided of 18 hours per week [2]. Hence, informal caregivers form an essential component of the healthcare system, reducing healthcare delivery costs in the US.

The physical and mental well-being of the informal caregiver is important, and research into understanding and improving the health of this population is necessary. Tasks related to caregiving often limit informal caregivers from carrying out activities of their interest or responsibilities at their wage-related job. Some informal caregivers, additionally, have to deal with challenging behavioral problems of the care recipient, such as verbal or physical aggression and confusion. Caregiving can be incrementally taxing for informal caregivers with physical, mental, and financial consequences. A scoping review and meta-analysis on the effects of caregiving revealed that informal caregivers report higher levels of stress/distress, depression, emotional problems, cognitive problems, poor sleep quality, and exacerbation of pre-existing chronic conditions compared to non-caregivers [3–7]. A longitudinal study of caregiver effects found that caregivers have increased psychological burden and decreased well-being over time [8]. Quality of life (QOL) offers a broader perspective in terms of the impact of life stressors, including caregiving, on physical, psychological, social, and financial well-being [9–14]. Previous studies that measured QOL found that informal caregiving is associated with lower scores on mental health, social functioning, and economic well-being compared to non-caregivers[10–12].

There is considerable variation among informal caregivers in the experience of burden and QOL, even if they are similar in clinical aspects, patient condition, or intensity of caregiving [11]. Psycho-social factors like stress appraisal, coping responses, social support, and the relationship between caregiver and care recipient have been found to explain such variation [15]. For example, informal caregivers of patients with Alzheimer’s for whom caregiving tasks are not as stressful, had greater social support, or better coping responses, such as problem-focused coping rather than emotion-focused coping, were found to experience lesser burden [16]. As a result, researchers have developed and tested models to explain the subjective variation in stress and provide a better understanding of poor health outcomes.

The Transactional Stress Theory developed by Lazarus and Folkman proposes that an outcome experienced by an individual depends on their appraisal stressors and coping strategy implemented as a result of the stressor [17]. Previous studies have provided evidence in support of this theory in caregivers of patients with multiple sclerosis, patients with left ventricular assist device, and children during COVID-19 pandemic [18–20]. The model demonstrates that the effect of caregiver-perceived stress on caregiver outcomes is mediated through the type of coping behavior adopted (i.e., positive or negative, adaptive or maladaptive). For example, a study testing the mediation analysis of stress-coping-depressive symptoms reported that personal mastery of stressors, coping efficacy, social or recreational activity restriction, and avoidance coping strategies, mediate the relationship between stressor and depressive symptoms where personal mastery and coping efficacy (both examples of positive coping) decreased depressive symptoms while avoidance coping and activity restriction (both examples of negative or maladaptive coping) increased depressive symptoms[21].

The outcomes utilized in these studies testing the mediation effect focus on narrow measures like caregivers’ depression, life satisfaction, and self-reported health. There is less focus on QOL, a construct which allows for an improved understanding of the association of stressors and coping mechanisms with social and economic well-being as well as physical and mental function. In addition, little is known whether the stress appraisal and the coping mechanism adopted are similar for caregivers providing care to patients differing in age or disease condition, e.g., older patients versus children.

## Methods

### Aims and study design

The current study aimed to understand the appraisal process of QOL, specifically evaluating how coping strategies mediate the relationship between stress and QOL among informal caregivers, and comparing informal caregivers of older adults and informal caregivers of children with respect to this process using a cross-sectional survey.

### Study sample

The caregiver groups selected to participate were informal caregivers of patients with either Alzheimer’s disease (AD) or Autism Spectrum Disorder (ASD). These groups were selected in order to have one group that was specifically providing care to older people and another group that was specifically providing care to children as each group can experience distinct caregiving challenges. We selected informal caregivers of patients with AD because the incidence of this condition is higher in older populations, and previous research showed that the caregiver burden is worse compared to caregivers of other neurological conditions [22]. We selected informal caregivers of patients with ASD because these caregivers experience a greater degree of burden compared to caregivers of children with other developmental disorders or mental health conditions [23]. A national convenience sample was obtained from Rare Patient Voice LLC, Towson, Maryland, a market research vendor company, that maintains panels of patients and caregivers in various conditions. Caregivers were eligible to participate in the study if they were adults (≥18 years of age) and provided unpaid care either to an older (≥65 years) patient with AD or a child ( < 18 years) with ASD.

### Survey design and distribution

Informal caregivers who met the inclusion criteria were sent a cover letter and a URL link to the survey programmed in Qualtrics (Qualtrics Inc, Provo, UT) via email. The survey included previously-developed instruments pertinent to the study, including perceived stress, coping behavior, QOL, and several questions about socio-demographic characteristics. The survey link was open for participants for 3 months, starting from the date of the first email. Reminders were sent bi-weekly to secure the maximum number of respondents to the survey. All respondents were asked to consent to participate and were provided with an honorarium for participating in the study. The study received approval from the Institutional Review Board of the University of Mississippi.

### Study measures

World Health Organization Quality of Life – Brief (WHOQOL-BREF): The WHOQOL-BREF is a brief version of the WHOQOL-100 which was developed and validated across 23 countries [24]. The WHOQOL-BREF has 26 items, including one item from each of 24 facets of QOL, one item on overall QOL, and one on general health. The 24 items representing each facet of QOL are measured using a five-point response format. The items are classified into four domains: physical health (seven items), psychological (six items), social relationships (three items), and environment (eight items). The raw scores for each domain were calculated by adding the scores of the items in each domain and these raw scores were transformed to a 4–20 scale using the algorithm provided by the developers of the WHOQOL-BREF [25]. Lower scores on the scale indicate poorer QOL.

Perceived stress scale: The overall stress levels of the informal caregiver were calculated by the Perceived Stress Scale (PSS), a 14-item instrument. It is designed to evaluate the degree of stress the individual perceives while facing specific life situations [26]. Specifically, it asks subjects how often they have had particular thoughts or feelings during the past month. It uses a five-point response format ranging from “Almost never” to “Very often”. Scores on PSS range from 0 to 56, with higher scores indicating more perceived stress.

Brief Coping Orientation to Problem Experience (Brief COPE): The coping behaviors used by informal caregivers were determined using the Brief Coping Orientation to Problem Experience (Brief COPE) measure [27]. Brief COPE is a 28–item instrument with each item having a four-point response from “Not doing it at all” to “Doing it a lot”. Based on prior research with caregivers of individuals with dementia, the items were divided into three sub-scales: emotion-focused, problem-focused, and dysfunctional coping [28]. Emotion-focused coping is measured by items that ask about their use of strategies of acceptance, emotional support, humor, positive reframing, and religion [28]. Problem-focused coping is measured by items representing active coping, instrumental support, and planning, while dysfunctional coping is measured using items representing self-distraction, denial, venting, behavioral disengagement, and self-blame [28]. Item scores were summed to determine the total score on emotion-focused, problem-focused, and dysfunctional coping, with higher scores indicating more frequent use of coping behaviors.

Demographic and caregiving information: Information was collected on the following socio-demographic and caregiving characteristics: (1) age, (2) race/ethnicity, (3) sex of the caregiver, (4) sex of the care recipient, (5) marital status, (6) occupational status, (7) education status, (8) relationship to care recipient, (9) presence of chronic conditions for the caregiver, (10) year caregiving started, and (11) number of caregiving hours per week.

### Data quality and statistical analysis

**Fig. 1:**
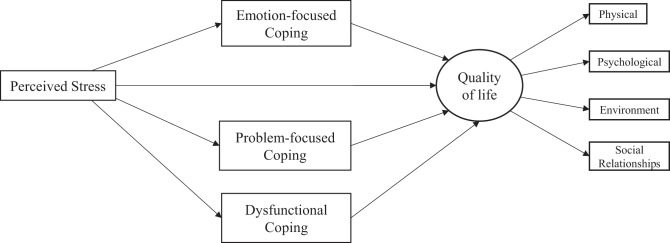
The caregiver stress-coping-quality of life model based on the Transactional Stress Theory. *Notes*: All paths were adjusted for potential confounding variables. Differences in model paths, including direct effects and indirect effects, between informal caregivers of older adult patients with Alzheimer’s disease and informal caregivers of children with autism spectrum disorder were assessed using multiple-group structural equation modeling in Mplus

Caregivers with complete and valid responses to all measures were included in the analyses. The quality of responses was assessed by examining respondents’ pattern of straight-lining (i.e., consistent response on more than or equal to half the items in a scale) or quick response times (i.e., total response time less than the cut-off for completing the entire survey applying a rule-of-thumb of 2 seconds per item). Respondents exceeding thresholds were deleted according to guidance provided by Huang et al [29]. All study measures were summarized using descriptive statistics. Internal consistency reliability was calculated for all scales using Cronbach’s alpha. A Cronbach’s alpha of ≥ 0.70 was indicative of good internal consistency reliability. Structural equation modeling (SEM) was used to test the mediating role of coping behavior in the perceived stress and QOL relationship. A parallel multiple mediator approach, as outlined by Hayes [30], was employed to estimate and test the hypothesized relationships among the variables after controlling for caregiver demographic characteristics (age, sex, race, ethnicity, marital status, education level, occupation, region, residence area, and relationship to patient), patient demographic characteristics (sex), and caregiving characteristics (presence of chronic conditions, formal service use, caregiving hours per week) [see Fig.1]. Perceived stress was the antecedent variable and was modeled as a global construct with total scale scores used in the analysis (i.e., each participant’s responses were summed across all 14 items). Global QOL was the outcome variable and was modeled as a latent variable derived from the scores on the four individual domains of the WHOQOL-BREF (i.e., domain scores served as indicators of a latent variable, similar to the approach used by Lanfredi et al [31]. and Johansen et al. [32]). In addition, individual subscale scores measuring the psychological, physical health, social relationships, and environment domains were used as separate dependent variables in subsequent SEM analyses to assess whether the tested effects differed across the multiple QOL domains. Coping, as measured by the Brief COPE, had scores for emotion-focused, problem-focused, and dysfunctional coping obtained by summing individual items representing the three constructs; these three variables were the mediators in the tested model. When using SEM, it is recommended to have a sample size of at least 200 [33], therefore, an a priori sample size of at least 200 for both groups of informal caregivers (patients with ASD or patients with AD) was considered adequate.

The parallel mediation model was assessed for both groups – caregivers of patients with AD and caregivers of patients with ASD – separately to derive estimates that indicated the linear relationship between the included variables (i.e., estimates of the individual paths between variables as well as indirect effects). The strength of the estimated relationships depended on the size of the value of the estimate, where values closer to zero signified weaker relationships and values further from zero signified stronger relationships. Multiple-group analysis was carried out to assess whether the estimates (i.e., individual paths as well as direct and indirect effects) of the parallel multiple mediator model differed between the two caregiver groups. All analyses were conducted in Mplus version 8 (Muthén & Muthén, Los Angeles, CA).

## Results

The panel used for the study had 1,239 caregivers, which included 798 caregivers of patients with AD and 441 caregivers of patients with ASD. A total of 574 caregivers responded to the survey, giving a response rate of 46.3%. Out of the 574 who responded, 140 were removed based on incomplete responses on one or more scales, while 17 respondents were removed based on invalid responses determined from the response quality assessment. Out of 417 included in the analysis, 210 (50.4%) were caregivers of patients with AD, and 207 (49.6%) were caregivers of patients with ASD. Most of the caregivers were either a son or a daughter of the patients (50.5%) in the AD group, while in the ASD group, most caregivers were parents (88.4%). A large proportion of the caregivers in both groups were females, White, 31–45 years of age, were married, had some college education, employed, were from the Southern U.S., or lived in a suburban area (Table 1). More than half of the caregivers had no chronic condition, and around two-thirds had used some formal service for caregiving. The Cronbach’s alpha of the WHOQOL-BREF was 0.94, PSS was 0.83, emotion-focused coping domain was 0.77, problem-focused coping domain was 0.79, and dysfunctional coping domain was 0.79.

**Table 1:** Demographic characteristics of the caregivers responding to the survey and included in the final analysis

	Informal caregivers of patients with AD (N = 210)	Informal caregivers of patients with ASD (N = 207)
**Relation to the patient**		
Spouse	24 (11.4%)	2 (1.0%)
Parent	5 (2.4%)	183 (88.4%)
Son/Daughter	106 (50.5%)	1 (0.5%)
Grandparent	3 (1.4%)	4 (1.9%)
Sibling	3 (1.4%)	4 (1.9%)
Friend	27 (12.9%)	7 (3.4%)
Other	42 (20%)	6 (2.8%)
**Age category of caregiver**		
18–30 years	34 (16.2%)	31 (15.0%)
31–45 years	112 (53.3%)	153 (73.9%)
45 years and above	64 (30.5%)	23 (11.1%)
Age in years, Mean (SD)	41 (12.6)	37 (6.6)
**Caregiver sex**		
Male	13 (6.2%)	20 (9.7%)
Female	197 (93.8%)	187 (90.3%)
**Patient sex**		
Male	78 (37.1%)	142 (68.6%)
Female	132 (62.9%)	65 (31.4%)
**Race of the caregiver**		
White	148 (70.5%)	168 (81.2%)
African American	20 (9.5%)	20 (9.7%)
Hispanic	33 (15.7%)	13 (6.3%)
Other	9 (4.3%)	6 (2.9%)
**Ethnicity of the caregiver**		
Hispanic	33 (15.7%)	13 (6.3%)
Non-Hispanic	177 (84.3%)	194 (93.7%)
**Marital status of the caregiver**		
Married	108 (51.4%)	121 (58.4%)
Widowed/Divorced/Separated	41 (19.5%)	37 (17.9%)
Never Married	61 (29.1%)	49 (23.7%)
**Education level of the caregiver**		
High school graduate or less	53 (25.2%)	43 (20.8%)
Some college	61 (29.0%)	71 (34.3%)
4-year or 2-year degree	73 (34.8%)	72 (34.8%)
Professional degree	23 (11.0%)	21 (10.1%)
**Occupation of the caregiver**		
Employed/Self-employed full time	70 (33.3%)	69 (33.3%)
Employed part-time	39 (18.6%)	31 (15.0%)
Unemployed, looking for work	16 (7.6%)	12 (5.8%)
Unemployed, not looking for work	8 (3.8%)	4 (1.9%)
Retired	27 (12.9%)	3 (1.4%)
Student	5 (2.4%)	4 (1.9%)
Home-make	39 (18.6%)	75 (36.2%)
Other	6 (2.9%)	9 (4.3%)
**Residential area of the caregiver**		
Urban	60 (28.6%)	47 (22.6%)
Suburban	77 (36.6%)	98 (47.4%)
Rural	73 (34.8%)	62 (30.0%)
**Region of residence of the caregiver**		
Northeast	25 (11.9%)	36 (17.4%)
Midwest	19 (9.0%)	49 (23.7%)
South	124 (59.0%)	85 (41.1%)
West	34 (16.3%)	30 (14.6%)
No response	8 (3.8%)	7 (3.4%)
**Does caregiver have any chronic condition?**		
Yes	88 (41.9%)	98 (47.3%)
No	122 (58.1%)	109 (52.7%)
**Has caregiver used any formal service**^**a**^?		
Yes	145 (69.0%)	146 (70.5%)
No	65 (31.0%)	61 (29.5%)
**Caregiving hours per week, mean (SD)**	47.8 (31.9)	93.4 (51.7)
**Perceived stress score**^**b**^**, mean(SD)**	27.7 (8.2)	27.6 (7.8)
**Quality of life**^**c**^**, mean (SD)**		
Physical health	13.8 (3.1)	13.3 (2.99)
Psychological	13.4 (3.42)	12.8 (3.33)
Social relationships	12.4 (3.86)	11.9 (4.04)
Environment	14.2 (2.59)	13.5 (2.72)

^a^Formal service included respite/day care, caregiver training or educational sessions, support groups in the community

^b^Scores on PSS range from 0 to 56 with higher score indicating more perceived stress

^c^Raw scores on each domain were transformed to a scale of 4–20 with low score indicating poor QOL

The direct effect of perceived stress on global QOL was significant for both groups (estimate = −0.601, 95% bootstrap [BS] confidence interval [CI] = −0.731 to −0.468 for the AD group and estimate = −0.637, 95% CI = −0.777 to −0.529 for the ASD group), signifying a decrease in QOL with an increase in perceived stress in caregiving (Table 2). Individual paths from perceived stress to coping were significant for both sets of caregivers. An increase in perceived stress significantly decreased the use of emotion-focused coping in the ASD group (estimate = −0.414, 95% CI = −0.535 to −0.249). An increase in perceived stress significantly decreased the use of problem-focused coping only in caregivers of patients with ASD (estimate = −0.327, 95% CI = −0.46 to −0.144). An increase in perceived stress significantly increased the use of dysfunctional coping in both groups (estimate = 0.654, 95% CI = 0.537 to 0.756 for the AD group and estimate = 0.44, 95% CI = 0.287 to 0.529 for the ASD group). However, with respect to associations between coping strategies and QOL, only the use of dysfunctional coping in caregivers of patients with AD was significantly associated with QOL (estimate = −0.139, 95% CI = −0.273 to −0.001).

**Table 2:** Estimates from individual parallel multiple mediator models and comparison of model estimates between informal caregivers

Path from the model	Estimate	SE	95% LLCI	95% ULCI
**Caregivers of patients with Alzheimer’s disease**				
Perceived stress → Emotion-focused coping	−0.134	0.08	−0.289	0.018
Perceived stress → Problem-focused coping	−0.062	0.077	−0.215	0.087
Perceived stress → Dysfunctional coping	0.654	0.055	0.537	0.756
Emotion-focused coping → Quality of life^a^	0.068	0.077	−0.087	0.214
Problem-focused coping → Quality of life^a^	0.102	0.078	−0.048	0.256
Dysfunctional coping → Quality of life^a^	−0.139	0.07	−0.273	−0.001
Indirect effect 1: Perceived stress → Emotion-focused coping → Quality of life^a^	−0.003	0.004	−0.012	0.001
Indirect effect 2: Perceived stress → Problem-focused coping → Quality of life^a^	−0.002	0.003	−0.01	0.001
Indirect effect 3: Perceived stress → Dysfunctional coping → Quality of life^a^	−0.026	0.013	−0.048	−0.005
Total indirect effect (i.e., all mediators)	−0.03	0.014	−0.053	−0.007
Direct effect (i.e., Perceived stress → Quality of life^a^)	−0.601	0.066	−0.731	−0.468
**Caregivers of patients with Autism**				
Perceived stress → Emotion-focused coping	−0.414	0.072	−0.535	−0.249
Perceived stress → Problem-focused coping	−0.327	0.079	−0.46	−0.144
Perceived stress → Dysfunctional coping	0.44	0.061	0.287	0.529
Emotion-focused coping → Quality of life^a^	0.101	0.069	−0.034	0.238
Problem-focused coping → Quality of life^a^	0.042	0.06	−0.07	0.165
Dysfunctional coping → Quality of life^a^	−0.09	0.065	−0.22	0.034
Indirect effect 1: Perceived stress → Emotion-focused coping → Quality of life^a^	−0.013	0.01	−0.032	0.002
Indirect effect 2: Perceived stress → Problem-focused coping → Quality of life^a^	−0.004	0.007	−0.017	0.006
Indirect effect 3: Perceived stress → Dysfunctional coping → Quality of life^a^	−0.012	0.01	−0.03	0.004
Total indirect effect (i.e., all mediators)	−0.03	0.016	−0.056	−0.004
Direct effect (i.e., Perceived stress → Quality of life^a^)	−0.637	0.065	−0.777	−0.529
**Difference between effect estimates in the Autism and Alzheimer’s disease models**^**b**^				
Perceived stress → Emotion-focused coping	0.213	0.08	0.079	0.341
Perceived stress → Problem-focused coping	0.144	0.056	0.045	0.231
Perceived stress → Dysfunctional coping	0.169	0.064	0.062	0.273
Emotion-focused coping → Quality of life^a^	−0.016	0.042	−0.087	0.051
Problem-focused coping → Quality of life^a^	0.036	0.06	−0.061	0.14
Dysfunctional coping → Quality of life^a^	−0.013	0.04	−0.079	0.051
Perceived stress → Emotion-focused coping → Quality of life^a^	0.011	0.011	−0.007	0.029
Perceived stress → Problem-focused coping → Quality of life^a^	0.003	0.008	−0.01	0.015
Perceived stress → Dysfunctional coping → Quality of life^a^	−0.013	0.017	−0.04	0.014
Total indirect effect	0	0.021	−0.035	0.035
Direct effect	0.032	0.029	−0.016	0.08

Note: All estimates are derived after controlling for potential confounding variables. All estimates are completely standardized. Lower limit (LL) and upper limit (UL) of the confidence interval (CI) are based on 95% percentile bootstrap confidence intervals

^a^ Quality of life was measured as a latent variable with each of the four domain scores from the WHOQOL-BREF serving as indicators

^b^ Difference testing assessed whether the path differed in magnitude between the Autism and Alzheimer’s disease model

The results of tests of parallel mediation and the corresponding differences in indirect and direct effects between the two groups are given in Table 2. The indirect effect from perceived stress to dysfunctional coping to global QOL was significant (estimate = −0.026, CI = −0.048 to −0.005) only for the AD group. All other indirect effects were non-significant. The total indirect effect, which is the sum of individual indirect effects, was significant for caregivers of patients with AD (estimate = −0.03, BS 95% CI = −0.053 to −0.007) as well as for caregivers of patients with ASD (estimate = −0.03, BS 95% CI = −0.056 to −0.004); this effect was mostly driven by the negative indirect effect associated with dysfunctional coping (i.e., greater stress was associated with more dysfunctional coping, which was in turn associated with lower QOL).

Multiple-group testing showed a significant difference between the two caregiver groups regarding how perceived stress was associated with emotion-focused, problem-focused, and dysfunctional coping. The associations between perceived stress and emotion-focused and problem-focused coping were more pronounced (i.e., negative) among caregivers of patients with ASD compared to caregivers of patients with AD, while the perceived stress and dysfunctional coping association was larger (i.e., more positive) for caregivers of patients with AD. However, because the coping strategies relationships with QOL were not significantly different between the groups (and generally quite small overall), none of the differences in indirect effects between the two caregiver groups were found to be statistically significant. The groups also did not differ significantly with respect to the direct effect of perceived stress on QOL. The results were consistent when individual domains of QOL were analyzed as outcomes (Supplemental materials).

## Discussion

Results from this cross-sectional study further improve our understanding of the appraisal process for QOL among caregivers and compares this process among two sets of caregivers who differed in the type of patient for whom they were providing care: Older patients with AD and children with ASD. The Transactional Stress Theory developed by Lazarus and Folkman [17] was used as a guide to understand the role of perceived stress on caregiver’s QOL among each set of caregivers, and explore how coping strategies of these caregivers may play a role in caregiver’s stress and QOL. The study found that perceived stress was negatively associated with QOL among both sets of caregivers. The negative effect of perceived stress persisted in each of the four domains of WHOQOL-BREF, i.e., physical health, psychological, social relationships, and environment. This finding is consistent with the results of previous studies conducted with caregivers of patients with AD and patients with ASD, which have independently found a significant association of perceived stress on QOL or family QOL [34, 35]. Comparing the effects of perceived stress on QOL between caregivers of patients with AD and patients with ASD showed no difference in the strength of the association, suggesting that the effect of stress on QOL is similar in both sets of caregivers. Carona et al. explored the association between caregiver burden and QOL among two groups of caregivers caring for children with either epilepsy or cerebral palsy and found the association was invariant between the two groups[36]. The type of condition, therefore, does not appear to independently influence the relationship between caregiver stress and QOL. When considering strategies to improve the QOL of caregivers, addressing sources of stress should be the primary focus irrespective of patient condition or age.

Testing and comparing the parallel multiple mediator model in the current study found that dysfunctional coping mediated the association between perceived stress and QOL among caregivers of patients with AD, meaning that the negative effect of perceived stress on QOL was more pronounced when caregivers used dysfunctional coping strategies. However, the effect of stress on QOL was unaffected by the use of emotion-focused or problem-focused coping strategies among these caregivers. For caregivers of children with ASD, the effect of perceived stress on their QOL was unaffected by any coping mechanisms utilized. This is contrary to the results of previous studies that tested the mediation effect of coping strategies in caregivers, showing mediation of coping strategies between stress and outcome [16, 21, 36, 37]. The difference in findings may be attributed to the differences in the sample of caregivers, type of coping strategies assessed, and the instruments utilized to measure coping and outcome. The total indirect effect, however, was significant for both groups, suggesting some mediating role for coping strategies between perceived stress and QOL among both sets of caregivers.

The results related to non-significant mediation by the individual coping strategies among caregivers of patients with ASD are most likely because of finding that these coping strategies had no significant associations with QOL. A couple of studies, one conducted in a sample of caregivers providing care to children with autism and the other among caregivers of children with cerebral palsy, also found no effect of coping strategies on QOL [38, 39]. The authors suggest that adaptation to the child’s condition coupled with the non-progressive nature of the condition might have contributed to no effect of coping strategies. A similar assumption could be made for caregivers of children with ASD in the current study. Among caregivers of patients with AD, dysfunctional coping was significantly associated with QOL, while emotional-focused and problem-focused coping had no association, which corresponds with the findings of Muscat and Scerri [40]. The higher stress levels among caregivers compared to population norms (PSS score of 27.7 vs. 19.6 in normal population [41]) might have limited the effect of coping mechanisms on QOL.

This is the first study to test whether there is a difference in the appraisal process of QOL between two distinct groups of caregivers: Those providing care to older adults with AD and those providing care to children with ASD. The study findings demonstrate that the indirect effects of perceived stress through coping mechanisms on QOL, were not significantly different between the two groups of caregivers. Previous studies have explored comparisons of the stress process between Black and White family caregivers of patients with AD and between caregiving husbands and wives, reporting similarities in the stress and coping mechanisms between the caregiving groups [16, 42]. This highlights the widely applicable nature of the Transactional Stress Model and suggests that understanding individual predictors of QOL is important to plan caregiver-specific interventions. The results from this study can help to drive future research on QOL by looking at how psycho-social factors like self-efficacy and social support play a role in the appraisal of QOL. Also, as the number of caregivers is likely to increase, the implications of age on QOL and how it fits into the theoretical model can be further explored.

Currently, the number of informal caregivers in the US far exceeds the number of paid resources available to help patients. As the number of patients with disabling chronic conditions is projected to increase, informal caregivers will play an even more significant role in fulfilling the requirement for providing care to these patients. Therefore, it is crucial for policymakers to provide enough support to caregivers in order to manage stress levels and maintain the availability of formal services. Caregivers may benefit from information about effective programs like National Family Caregiver Support Program, Veteran Affairs Caregiver Support Program or North Carolina Family Caregiver Support Program that provide training to manage a patient’s condition, caregiver stress, and financial burden. Mental health professionals can also collaborate with providers of patients, ensuring ease in referrals for their caregivers or collaborate with local public health departments to offer awareness campaigns on availability of supportive services and useful resources for caregivers. Additionally, increasing financial and workplace protection for caregivers and increasing access to services and supports to assist caregivers would help to improve QOL [43]. All of the above strategies can mitigate caregiver stress, which was found to be an important predictor of QOL of caregivers in this study.

Some study limitations should be considered. A convenience sample of caregivers from a market research vendor was used to obtain responses for the study. The sample of caregivers may not represent the national sample of caregivers and therefore, any generalization of study results should be made with caution. This study may be subject to non-response bias as a number of respondents did not have complete responses to the online survey. Third, the segregation of the coping scale (Brief-COPE) into problem-focused, emotion-focused, and dysfunctional coping was based on previous research, but such a structure lacks extensive evidence from factor analysis. Future research should further evaluate the three-factor structure of the Brief-COPE rather than one focused only on maladaptive coping and adaptive coping (i.e., two factors). Finally, the severity of patients’ AD or ASD was not measured and might influence the relationship between stress and QOL. To minimize the impact of severity of patients’ condition on model estimates, number of caregiving hours per week was measured as a proxy and controlled for in the statistical analysis as severity of the condition has been shown to increase caregiving hours per week[44].

## Conclusion

Perceived stress of caregiving significantly affects the QOL of caregivers, and this relationship persists irrespective of whether caregivers are providing care to older adults with AD or children with ASD. Coping strategies adopted did not clearly alter the effect of stress on QOL among these caregivers except dysfunctional coping among caregivers of AD. Strategies focusing on reducing caregiving stress can significantly aid in improving caregivers’ QOL in these two patient populations.

## Electronic supplementary material

Below is the link to the electronic supplementary material.

Supplementary material 1

## Data Availability

The datasets used and/or analysed during the current study are available from the corresponding author on reasonable request.

## Declarations

### Previous presentations of the work

Poster presentation at American Pharmacists Association (APhA) Annual Meeting and Exposition 2024.

## Abbreviations

AD: Alzheimer’s Disease
ASD: Autism Spectrum Disorder
Brief COPE: Brief Coping Orientation to Problem Experience
BS: Bootstrap
CI: Confidence Interval
COVID-19: Coronavirus Disease 2019
LLC: Limited Liability Company
PSS: Perceived Stress Scale
QOL: Quality of Life
SEM: Structural equation modeling
US: United States
WHOQOL-BREF: World Health Organization Quality of Life – Brief
