# Supplementary material for: Does the relationship between stress and quality of life differ among informal caregivers of older adults with Alzheimer’s disease and children with autism spectrum disorder? Results from a cross-sectional survey
Source: J Patient Rep Outcomes. 2025 Oct 16;9:121. doi: 10.1186/s41687-025-00953-7 (PMC12532540; doi:10.1186/s41687-025-00953-7)
Supplement: Supplementary file 1 — Supplementary material 1 [file 41687_2025_953_MOESM1_ESM.docx]

Supplementary tables

| Table S1: Estimates from individual model and from comparison of parallel mediation models between Autism and Alzheimer’s caregivers for Physical Health as the main outcome | | | | |
| --- | --- | --- | --- | --- |
| Path from the model | Estimate | SE | LLCI | ULCI |
| Autism | | | | |
| Perceived stress → Emotion-focused coping | -0.414 | 0.072 | -0.535 | -0.249 |
| Perceived stress → Problem-focused coping | -0.327 | 0.079 | -0.46 | -0.144 |
| Perceived stress → Dysfunctional coping | 0.44 | 0.061 | 0.287 | 0.529 |
| Emotion-focused coping → Physical^a^ | -0.05 | 0.082 | -0.209 | 0.111 |
| Problem-focused coping → Physical^a^ | -0.013 | 0.07 | -0.149 | 0.123 |
| Dysfunctional coping → Physical^a^ | -0.002 | 0.073 | -0.136 | 0.156 |
| Perceived stress → Emotion-focused coping → Physical^a^ | 0.008 | 0.014 | -0.02 | 0.037 |
| Perceived stress → Problem-focused coping → Physical^a^ | 0.002 | 0.01 | -0.018 | 0.022 |
| Perceived stress → Dysfunctional coping → Physical^a^ | 0 | 0.014 | -0.026 | 0.029 |
| Total indirect effect | 0.01 | 0.022 | -0.034 | 0.052 |
| Direct effect | -0.537 | 0.08 | -0.691 | -0.378 |
| Alzheimer’s disease | | | | |
| Perceived stress → Emotion-focused coping | -0.134 | 0.08 | -0.289 | 0.018 |
| Perceived stress → Problem-focused coping | -0.062 | 0.077 | -0.215 | 0.087 |
| Perceived stress → Dysfunctional coping | 0.654 | 0.055 | 0.537 | 0.756 |
| Emotion-focused coping → Physical^a^ | -0.138 | 0.083 | -0.304 | 0.03 |
| Problem-focused coping → Physical^a^ | 0.168 | 0.084 | 0.002 | 0.332 |
| Dysfunctional coping → Physical^a^ | -0.141 | 0.072 | -0.28 | 0.003 |
| Perceived stress → Emotion-focused coping → Physical^a^ | 0.007 | 0.006 | -0.001 | 0.026 |
| Perceived stress → Problem-focused coping → Physical^a^ | -0.004 | 0.006 | -0.021 | 0.004 |
| Perceived stress → dysfunctional coping → Physical^a^ | -0.035 | 0.017 | -0.069 | 0 |
| Total indirect effect | -0.032 | 0.02 | -0.07 | 0.006 |
| Direct effect | -0.384 | 0.066 | -0.507 | -0.249 |
| Difference between each path of Autism and Alzheimer’s disease model^b^ | | | | |
| Perceived stress → Emotion-focused coping | 0.213 | 0.08 | 0.06 | 0.363 |
| Perceived stress → Problem-focused coping | 0.144 | 0.056 | 0.027 | 0.247 |
| Perceived stress → Dysfunctional coping | 0.169 | 0.064 | 0.042 | 0.294 |
| Emotion-focused coping → Physical^a^ | -0.046 | 0.062 | -0.171 | 0.074 |
| Problem-focused coping → Physical^a^ | 0.145 | 0.086 | -0.017 | 0.318 |
| Dysfunctional coping → Physical^a^ | -0.069 | 0.055 | -0.18 | 0.034 |
| Perceived stress → Emotion-focused coping → Physical^a^ | -0.001 | 0.016 | -0.033 | 0.03 |
| Perceived stress → Problem-focused coping → Physical^a^ | -0.006 | 0.011 | -0.029 | 0.015 |
| Perceived stress → Dysfunctional coping → Physical^a^ | -0.035 | 0.022 | -0.079 | 0.007 |
| Total indirect effect | -0.041 | 0.029 | -0.099 | 0.016 |
| Direct effect | 0.071 | 0.041 | -0.012 | 0.147 |

Note: All estimates are derived after controlling for potential confounding variables. All estimates are completely standardized. Lower limit (LL) and upper limit (UL) of the confidence interval (CI) are based on 95% percentile bootstrap confidence intervals.

1. Physical health was one of the four domains from the quality of life instrument used in the study (WHOQOL-BREF)
2. Difference testing assessed whether the path differed in magnitude between the Autism and Alzheimer’s disease model

| Table S2: Estimates from individual model and from comparison of parallel mediation models between Autism and Alzheimer’s caregivers for Psychological Health as the main outcome | | | | |
| --- | --- | --- | --- | --- |
| Path from the model | Estimate | SE | LLCI | ULCI |
| Autism | | | | |
| Perceived stress → Emotion-focused coping | -0.414 | 0.072 | -0.535 | -0.249 |
| Perceived stress → Problem-focused coping | -0.327 | 0.079 | -0.46 | -0.144 |
| Perceived stress → Dysfunctional coping | 0.44 | 0.061 | 0.287 | 0.529 |
| Emotion-focused coping → Psychological^a^ | 0.152 | 0.069 | 0.022 | 0.294 |
| Problem-focused coping → Psychological^a^ | 0.026 | 0.057 | -0.085 | 0.137 |
| Dysfunctional coping → Psychological^a^ | -0.105 | 0.064 | -0.237 | 0.017 |
| Perceived stress → Emotion-focused coping → Psychological^a^ | -0.028 | 0.015 | -0.061 | -0.002 |
| Perceived stress → Problem-focused coping → Psychological^a^ | -0.004 | 0.009 | -0.023 | 0.014 |
| Perceived stress → Dysfunctional coping → Psychological^a^ | -0.021 | 0.014 | -0.05 | 0.006 |
| Total indirect effect | -0.053 | 0.021 | -0.095 | -0.012 |
| Direct effect | -0.538 | 0.064 | -0.667 | -0.42 |
| Alzheimer’s disease | | | | |
| Perceived stress → Emotion-focused coping | -0.134 | 0.08 | -0.289 | 0.018 |
| Perceived stress → Problem-focused coping | -0.062 | 0.077 | -0.215 | 0.087 |
| Perceived stress → Dysfunctional coping | 0.654 | 0.055 | 0.537 | 0.756 |
| Emotion-focused coping → Psychological^a^ | 0.069 | 0.076 | -0.076 | 0.216 |
| Problem-focused coping → Psychological^a^ | 0.094 | 0.079 | -0.054 | 0.252 |
| Dysfunctional coping → Psychological^a^ | -0.102 | 0.074 | -0.244 | 0.047 |
| Perceived stress → Emotion-focused coping → Psychological^a^ | -0.004 | 0.006 | -0.021 | 0.003 |
| Perceived stress → Problem-focused coping → Psychological^a^ | -0.002 | 0.004 | -0.017 | 0.002 |
| Perceived stress → Dysfunctional coping → Psychological^a^ | -0.028 | 0.021 | -0.069 | 0.012 |
| Total indirect effect | -0.034 | 0.022 | -0.077 | 0.009 |
| Direct effect | -0.593 | 0.069 | -0.727 | -0.457 |
| Difference between each path of Autism and Alzheimer’s disease model^b^ | | | | |
| Perceived stress → Emotion-focused coping | 0.213 | 0.08 | 0.06 | 0.363 |
| Perceived stress → Problem-focused coping | 0.144 | 0.056 | 0.027 | 0.247 |
| Perceived stress → Dysfunctional coping | 0.169 | 0.064 | 0.042 | 0.294 |
| Emotion-focused coping → Psychological^a^ | -0.05 | 0.061 | -0.169 | 0.067 |
| Problem-focused coping → Psychological^a^ | 0.062 | 0.087 | -0.108 | 0.234 |
| Dysfunctional coping → Psychological^a^ | 0.007 | 0.059 | -0.106 | 0.125 |
| Perceived stress → Emotion-focused coping → Psychological^a^ | 0.024 | 0.016 | -0.005 | 0.058 |
| Perceived stress → Problem-focused coping → Psychological^a^ | 0.001 | 0.01 | -0.019 | 0.021 |
| Perceived stress → Dysfunctional coping → Psychological^a^ | -0.007 | 0.025 | -0.055 | 0.044 |
| Total indirect effect | 0.018 | 0.031 | -0.041 | 0.08 |
| Direct effect | -0.006 | 0.042 | -0.087 | 0.074 |

Note: All estimates are derived after controlling for potential confounding variables. All estimates are completely standardized. Lower limit (LL) and upper limit (UL) of the confidence interval (CI) are based on 95% percentile bootstrap confidence intervals.

1. Psychological health was one of the four domains from the quality of life instrument used in the study (WHOQOL-BREF)
2. Difference testing assessed whether the path differed in magnitude between the Autism and Alzheimer’s disease model

| Table S3: Estimates from individual model and from comparison of parallel mediation models between Autism and Alzheimer’s caregivers for Environment as the main outcome | | | | |
| --- | --- | --- | --- | --- |
| Path from the model | Estimate | SE | LLCI | ULCI |
| Autism | | | | |
| Perceived stress → Emotion-focused coping | -0.414 | 0.072 | -0.535 | -0.249 |
| Perceived stress → Problem-focused coping | -0.327 | 0.079 | -0.46 | -0.144 |
| Perceived stress → Dysfunctional coping | 0.44 | 0.061 | 0.287 | 0.529 |
| Emotion-focused coping → Environment^a^ | -0.043 | 0.079 | -0.207 | 0.108 |
| Problem-focused coping → Environment^a^ | 0.09 | 0.072 | -0.047 | 0.236 |
| Dysfunctional coping → Environment^a^ | 0.064 | 0.078 | -0.08 | 0.22 |
| Perceived stress → Emotion-focused coping → Environment^a^ | 0.006 | 0.012 | -0.016 | 0.033 |
| Perceived stress → Problem-focused coping → Environment^a^ | -0.011 | 0.01 | -0.034 | 0.005 |
| Perceived stress → Dysfunctional coping → Environment^a^ | 0.01 | 0.013 | -0.014 | 0.036 |
| Total indirect effect | 0.006 | 0.02 | -0.032 | 0.045 |
| Direct effect | -0.64 | 0.069 | -0.757 | -0.476 |
| Alzheimer’s disease | | | | |
| Perceived stress → Emotion-focused coping | -0.134 | 0.08 | -0.289 | 0.018 |
| Perceived stress → Problem-focused coping | -0.062 | 0.077 | -0.215 | 0.087 |
| Perceived stress → Dysfunctional coping | 0.654 | 0.055 | 0.537 | 0.756 |
| Emotion-focused coping → Environment^a^ | 0.097 | 0.088 | -0.077 | 0.266 |
| Problem-focused coping → Environment^a^ | 0.091 | 0.085 | -0.07 | 0.26 |
| Dysfunctional coping → Environment^a^ | -0.042 | 0.072 | -0.189 | 0.096 |
| Perceived stress → Emotion-focused coping → Environment^a^ | -0.004 | 0.005 | -0.02 | 0.002 |
| Perceived stress → Problem-focused coping → Environment^a^ | -0.002 | 0.003 | -0.014 | 0.002 |
| Perceived stress → Dysfunctional coping → Environment^a^ | -0.009 | 0.015 | -0.039 | 0.02 |
| Total indirect effect | -0.014 | 0.016 | -0.046 | 0.018 |
| Direct effect | -0.462 | 0.085 | -0.624 | -0.29 |
| Difference between each path of Autism and Alzheimer’s disease model^b^ | | | | |
| Perceived stress → Emotion-focused coping | 0.213 | 0.08 | 0.06 | 0.363 |
| Perceived stress → Problem-focused coping | 0.169 | 0.064 | 0.027 | 0.247 |
| Perceived stress → Dysfunctional coping | 0.144 | 0.056 | 0.042 | 0.294 |
| Emotion-focused coping → Environment^a^ | 0.063 | 0.055 | -0.047 | 0.167 |
| Problem-focused coping → Environment^a^ | 0 | 0.077 | -0.15 | 0.152 |
| Dysfunctional coping → Environment^a^ | -0.048 | 0.05 | -0.149 | 0.048 |
| Perceived stress → Emotion-focused coping → Environment^a^ | -0.011 | 0.014 | -0.038 | 0.015 |
| Perceived stress → Problem-focused coping → Environment^a^ | 0.009 | 0.01 | -0.009 | 0.031 |
| Perceived stress → Dysfunctional coping → Environment^a^ | -0.019 | 0.02 | -0.059 | 0.019 |
| Total indirect effect | -0.02 | 0.026 | -0.07 | 0.031 |
| Direct effect | 0.087 | 0.039 | 0.01 | 0.161 |

Note: All estimates are derived after controlling for potential confounding variables. All estimates are completely standardized. Lower limit (LL) and upper limit (UL) of the confidence interval (CI) are based on 95% percentile bootstrap confidence intervals.

1. Environment was one of the four domains from the quality of life instrument used in the study (WHOQOL-BREF)
2. Difference testing assessed whether the path differed in magnitude between the Autism and Alzheimer’s disease model

| Table S4: Estimates from individual model and from comparison of parallel mediation models between Autism and Alzheimer’s caregivers for Social Relationships as the main outcome | | | | | |
| --- | --- | --- | --- | --- | --- |
| Path from the model | Estimate | | SE | LLCI | ULCI |
| Autism | | | | | |
| Perceived stress → Emotion-focused coping | | -0.414 | 0.072 | -0.535 | -0.249 |
| Perceived stress → Problem-focused coping | | -0.327 | 0.079 | -0.46 | -0.144 |
| Perceived stress → Dysfunctional coping | | 0.44 | 0.061 | 0.287 | 0.529 |
| Emotion-focused coping → Social relationships^a^ | | 0.175 | 0.072 | 0.031 | 0.313 |
| Problem-focused coping → Social relationships^a^ | | 0.052 | 0.066 | -0.072 | 0.184 |
| Dysfunctional coping → Social relationships^a^ | | -0.23 | 0.065 | -0.373 | -0.116 |
| Perceived stress → Emotion-focused coping → Social relationships^a^ | | -0.039 | 0.019 | -0.08 | -0.006 |
| Perceived stress → Problem-focused coping → Social relationships^a^ | | -0.009 | 0.013 | -0.04 | 0.013 |
| Perceived stress → Dysfunctional coping → Social relationships^a^ | | -0.055 | 0.019 | -0.097 | -0.023 |
| Total indirect effect | | -0.103 | 0.029 | -0.163 | -0.05 |
| Direct effect | | -0.403 | 0.068 | -0.535 | -0.272 |
| Alzheimer’s disease | | | | | |
| Perceived stress → Emotion-focused coping | | -0.134 | 0.08 | -0.289 | 0.018 |
| Perceived stress → Problem-focused coping | | -0.062 | 0.077 | -0.215 | 0.087 |
| Perceived stress → Dysfunctional coping | | 0.654 | 0.055 | 0.537 | 0.756 |
| Emotion-focused coping → Social relationships^a^ | | 0.206 | 0.091 | 0.029 | 0.376 |
| Problem-focused coping → Social relationships^a^ | | -0.076 | 0.086 | -0.246 | 0.087 |
| Dysfunctional coping → Social relationships^a^ | | -0.224 | 0.081 | -0.385 | -0.067 |
| Perceived stress → Emotion-focused coping → Social relationships^a^ | | -0.013 | 0.011 | -0.043 | 0 |
| Perceived stress → Problem-focused coping → Social relationships^a^ | | 0.002 | 0.005 | -0.003 | 0.022 |
| Perceived stress → Dysfunctional coping → Social relationships^a^ | | -0.069 | 0.026 | -0.123 | -0.022 |
| Total indirect effect | | -0.079 | 0.027 | -0.136 | -0.027 |
| Direct effect | | -0.329 | 0.086 | -0.499 | -0.159 |
| Difference between each path of Autism and Alzheimer’s disease model^b^ | | | | | |
| Perceived stress → Emotion-focused coping | | 0.213 | 0.08 | 0.06 | 0.363 |
| Perceived stress → Problem-focused coping | | 0.144 | 0.056 | 0.027 | 0.247 |
| Perceived stress → Dysfunctional coping | | 0.169 | 0.064 | 0.042 | 0.294 |
| Emotion-focused coping → Social relationships^a^ | | 0.009 | 0.081 | -0.147 | 0.167 |
| Problem-focused coping → Social relationships^a^ | | -0.129 | 0.111 | -0.345 | 0.091 |
| Dysfunctional coping → Social relationships^a^ | | 0.03 | 0.072 | -0.118 | 0.169 |
| Perceived stress → Emotion-focused coping → Social relationships^a^ | | 0.026 | 0.022 | -0.016 | 0.071 |
| Perceived stress → Problem-focused coping → Social relationships^a^ | | 0.011 | 0.014 | -0.013 | 0.043 |
| Perceived stress → Dysfunctional coping → Social relationships^a^ | | -0.014 | 0.032 | -0.081 | 0.048 |
| Total indirect effect | | 0.024 | 0.041 | -0.057 | 0.105 |
| Direct effect | | 0.064 | 0.054 | -0.042 | 0.169 |

Note: All estimates are derived after controlling for potential confounding variables. All estimates are completely standardized. Lower limit (LL) and upper limit (UL) of the confidence interval (CI) are based on 95% percentile bootstrap confidence intervals.

1. Social relationships was one of the four domains from the quality of life instrument used in the study (WHOQOL-BREF)
2. Difference testing assessed whether the path differed in magnitude between the Autism and Alzheimer’s disease model
